# Supplementary material for: The 9p21.3 Coronary Artery Disease Risk Locus Modulates Vascular Cell-State Transitions via Enhancer-Driven Regulation of MTAP
Source: bioRxiv. 2025 Nov 18:2025.11.18.689066. Preprint. [Version 1] doi: 10.1101/2025.11.18.689066 (PMC12668014; doi:10.1101/2025.11.18.689066)
Supplement: Supplement 2 [file NIHPP2025.11.18.689066v1-supplement-2.pdf]

Figure S1

bioRxiv preprint doi: https://doi.org/10.1101/2025.11.18.689066; this version posted November 18, 2025. The copyright holder for this preprint (which was not certified by peer review) is the author/funder, who has granted bioRxiv a license to display the preprint in perpetuity. It is made available under aCC-BY 4.0 International license.

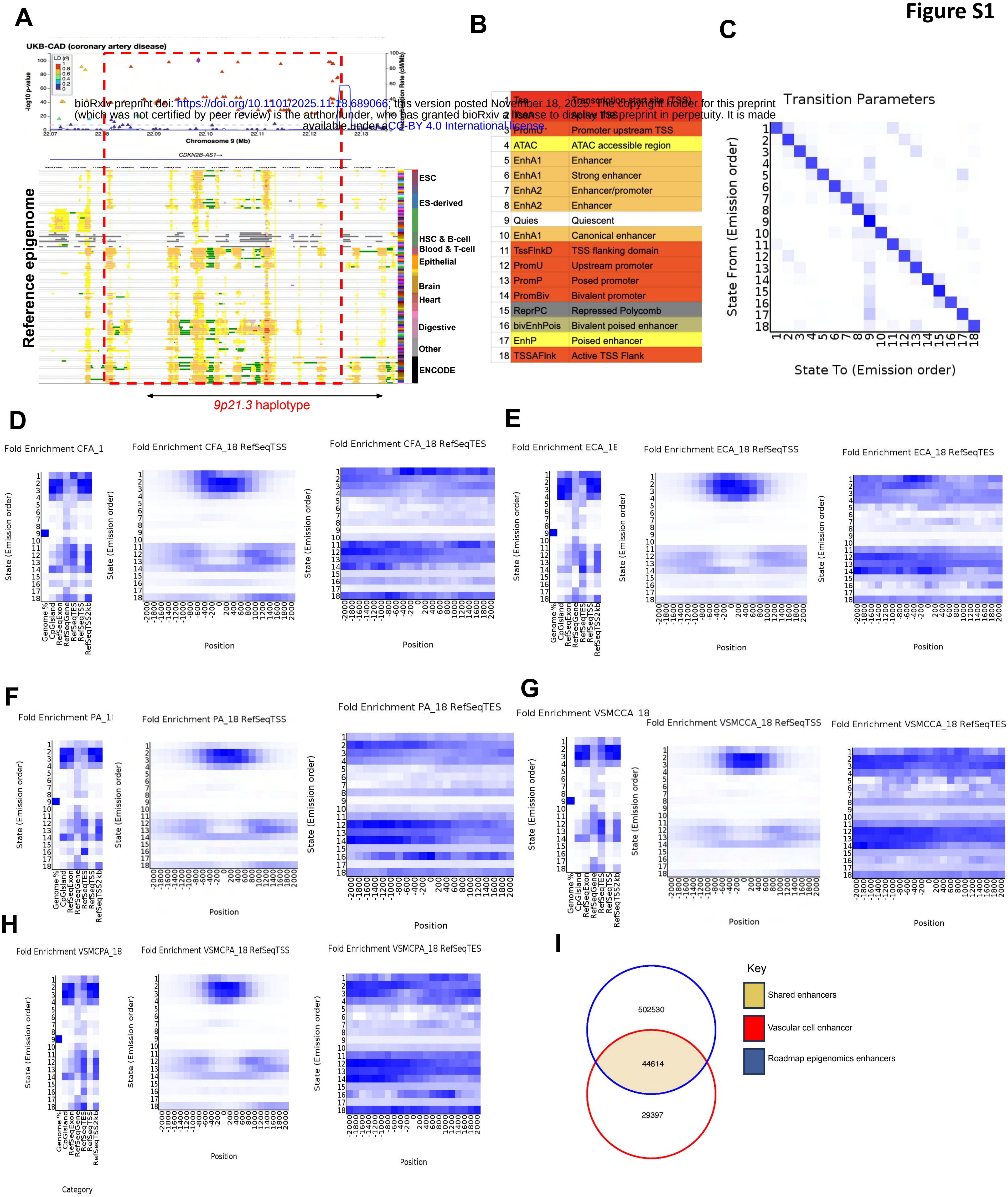

bioRxiv preprint doi: https://doi.org/10.1101/2025.11.18.689066; this version posted November 18, 2025. The copyright holder for this preprint (which was not certified by peer review) is the author/funder, who has granted bioRxiv a license to display the preprint in perpetuity. It is made available under aCC-BY 4.0 International license.

Figure S1. **Genome-wide chromatin state annotation of vascular cells by chromHMM**

(A) Publicly available chromatin state annotation using the ENCODE as well as Roadmap epigenome 111 available data sets. Overlaid above is the UKBB LocusZoom plot for CAD.

(B) Candidate state descriptions for each ChromHMM-annotated vascular cell chromatin state, with accompanying state abbreviation.

(C-H) Genomic annotations per cell type are displayed in distinct panels. The left heatmap illustrates the fold enrichment overlap for various genomic annotations. A darker blue indicates a higher fold enrichment, relative to a column-specific coloring scale. Next to this, a heatmap displays the fold enrichment for each state within 200-bp bins, spanning 2 kb around RefSeq transcription start sites (TSSs). A darker blue indicates greater fold enrichment, with a single color scale applied across the entire heatmap. The heatmap to the right shows the information but for transcript end sites.

(I) Venn diagram showing shared enhancers – with Roadmap epigenome project – and novel enhancers identified by chromHMM annotation of vascular cells

Figure S2

bioRxiv preprint doi: https://doi.org/10.1101/2025.11.18.689066; this version posted November 18, 2025. The copyright holder for this preprint (which was not certified by peer review) is the author/funder, who has granted bioRxiv a license to display the preprint in perpetuity. It is made available under aCC-BY 4.0 International license.

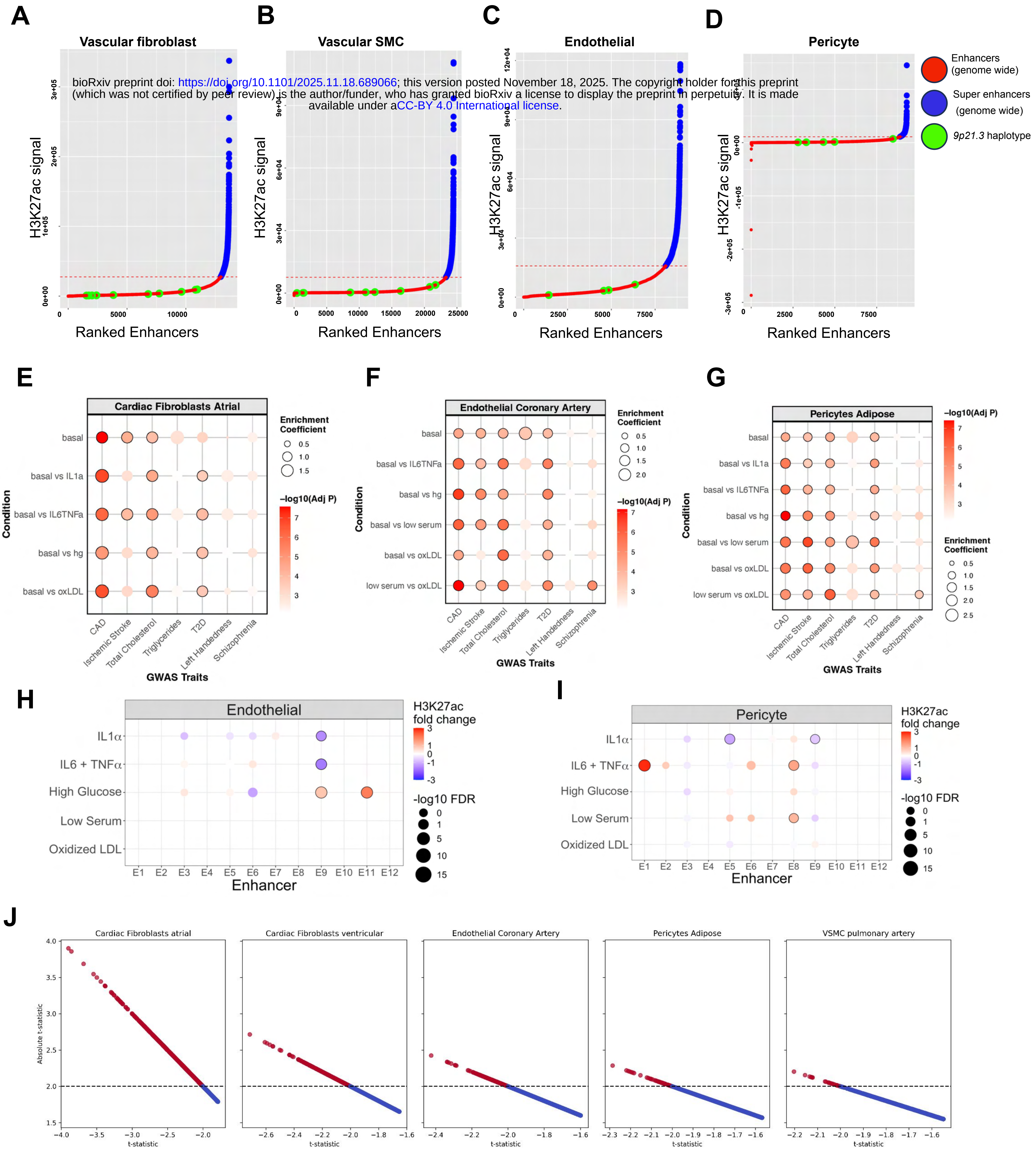

bioRxiv preprint doi: https://doi.org/10.1101/2025.11.18.689066; this version posted November 18, 2025. The copyright holder for this preprint (which was not certified by peer review) is the author/funder, who has granted bioRxiv a license to display the preprint in perpetuity. It is made available under aCC-BY 4.0 International license.

Figure S2. Characterization of *9p21.3* enhancers and identification of cell-types mediating risk for CAD

(A-D) Plots showing super enhancers (blue), enhancers (red), and enhancers within *9p21.3* haplotype (green).

(E-G) Dot plots showing enrichment for CAD heritability at baseline and in response to CAD-relevant stimulatory conditions, including Inflammatory (IL-1α and IL-6 & TNF-α) atrial derived fibroblast, coronary artery derived endothelial cells, and adipose derived pericytes. Solid dark lines around each dot represent significance at a threshold of $-\log_{10}$(adjusted P-value) ≥ 3.35.

(H-I) Dot plots showing the impact of CAD-relevant stimulatory conditions on enhancer activities (proxy of H3K27ac) at *9p21.3* locus in endothelial cells and pericytes. Fold changes are indicated as Red (increased fold change) or Blue (Reduced fold change) and solid dark lines around each dot represent significance at a threshold of $-\log_{10}$(FDR) ≥ 1.3.

(J) T-statistic versus absolute t-statistic (y-axis) for gene expression across five primary human vascular cell types under basal conditions. Differentially expressed genes, representing the top 10% by absolute t-statistic, are highlighted in red; all other genes are in blue. The dashed line indicates a high absolute t-statistic threshold.

Figure S3

bioRxiv preprint doi: https://doi.org/10.1101/2025.11.18.689066; this version posted November 18, 2025. The copyright holder for this preprint (which was not certified by peer review) is the author/funder, who has granted bioRxiv a license to display the preprint in perpetuity. It is made available under aCC-BY 4.0 International license.

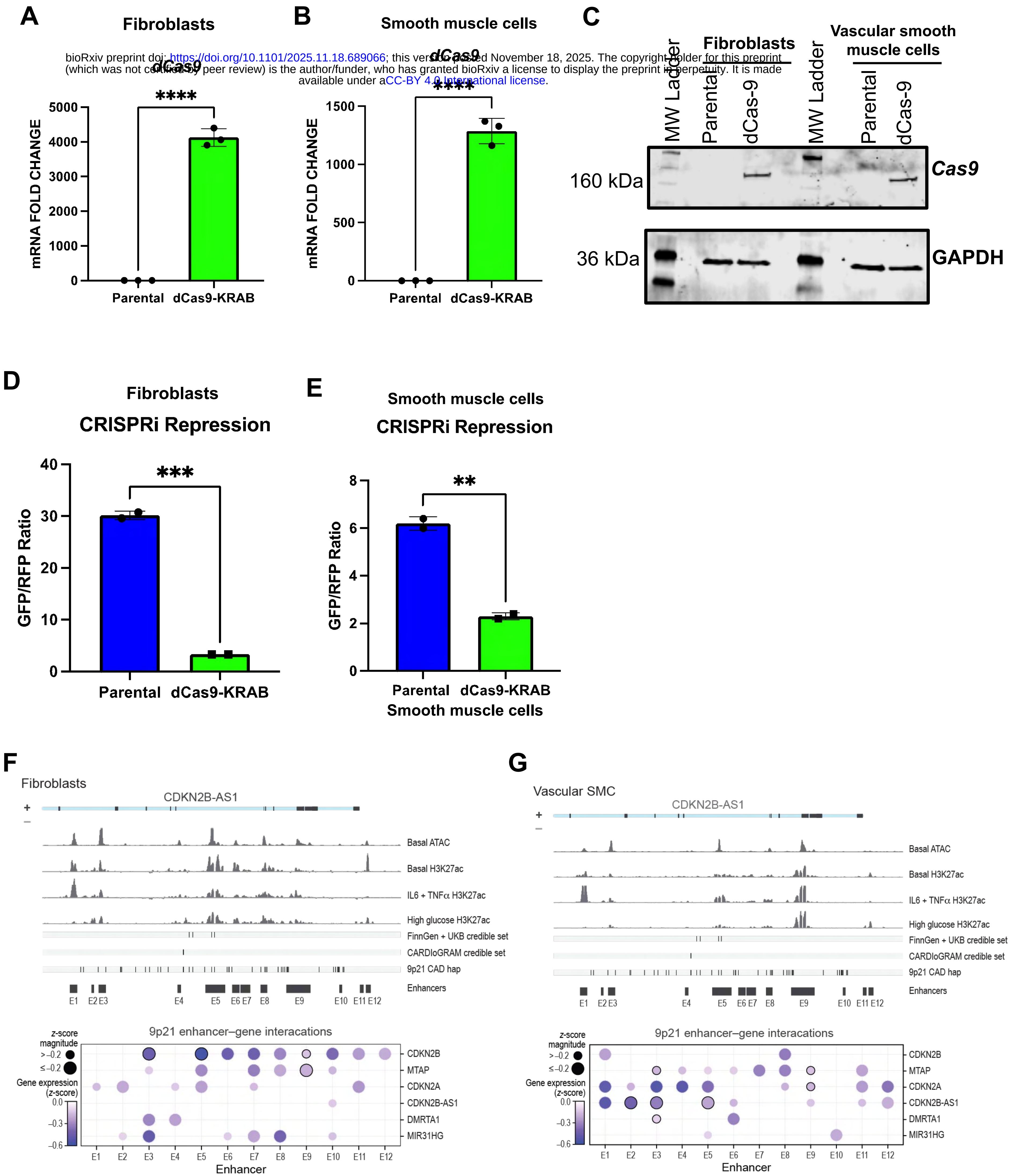

bioRxiv preprint doi: https://doi.org/10.1101/2025.11.18.689066; this version posted November 18, 2025. The copyright holder for this preprint (which was not certified by peer review) is the author/funder, who has granted bioRxiv a license to display the preprint in perpetuity. It is made available under aCC-BY 4.0 International license.

Figure S3. Generation of constitutive expressing dCa9-KRAB

(A-B) Bar graph showing RT-qPCR detection of dCas9-KRAB mRNA expression in both control parental cells and the constitutive dCas9-KRAB expressing vascular fibroblast (n=3) and smooth muscle cells (n=3). A t-test was used to identify significant differences between control vascular parental lines and dCas9-KRAB expressing vascular cells. P-value< 0.05 is considered significant.

(C) Western blot analysis showing detection of Cas9 protein in the dCas9-KRAB cell lines used in this study.

(D-E) Bar graphs showing dCas9-KRAB repression activities. A t-test was used to identify significant differences between control vascular parental lines and dCas9-KRAB expressing vascular cells. P-value<0.05 is considered significant.

(F-G) Dot plots showing aggregated effects of *9p21.3* enhancer guides on expression of cis-expressing genes in vascular fibroblast and smooth muscle cells. Dark border circles indicate independent signal validation by qPCR, which was conducted for enhancers E2, E3, E5, and E9. In this plot, we overlaid epigenetic enhancer signatures under basal and disease-relevant stimulatory conditions to show relative position of annotated *9p21.3* enhancers to epigenetic signatures around *9p21.3* haplotype.

Figure S4

bioRxiv preprint doi: https://doi.org/10.1101/2025.11.18.689066; this version posted November 18, 2025. The copyright holder for this preprint (which was not certified by peer review) is the author/funder, who has granted bioRxiv a license to display the preprint in perpetuity. It is made available under aCC-BY 4.0 International license.

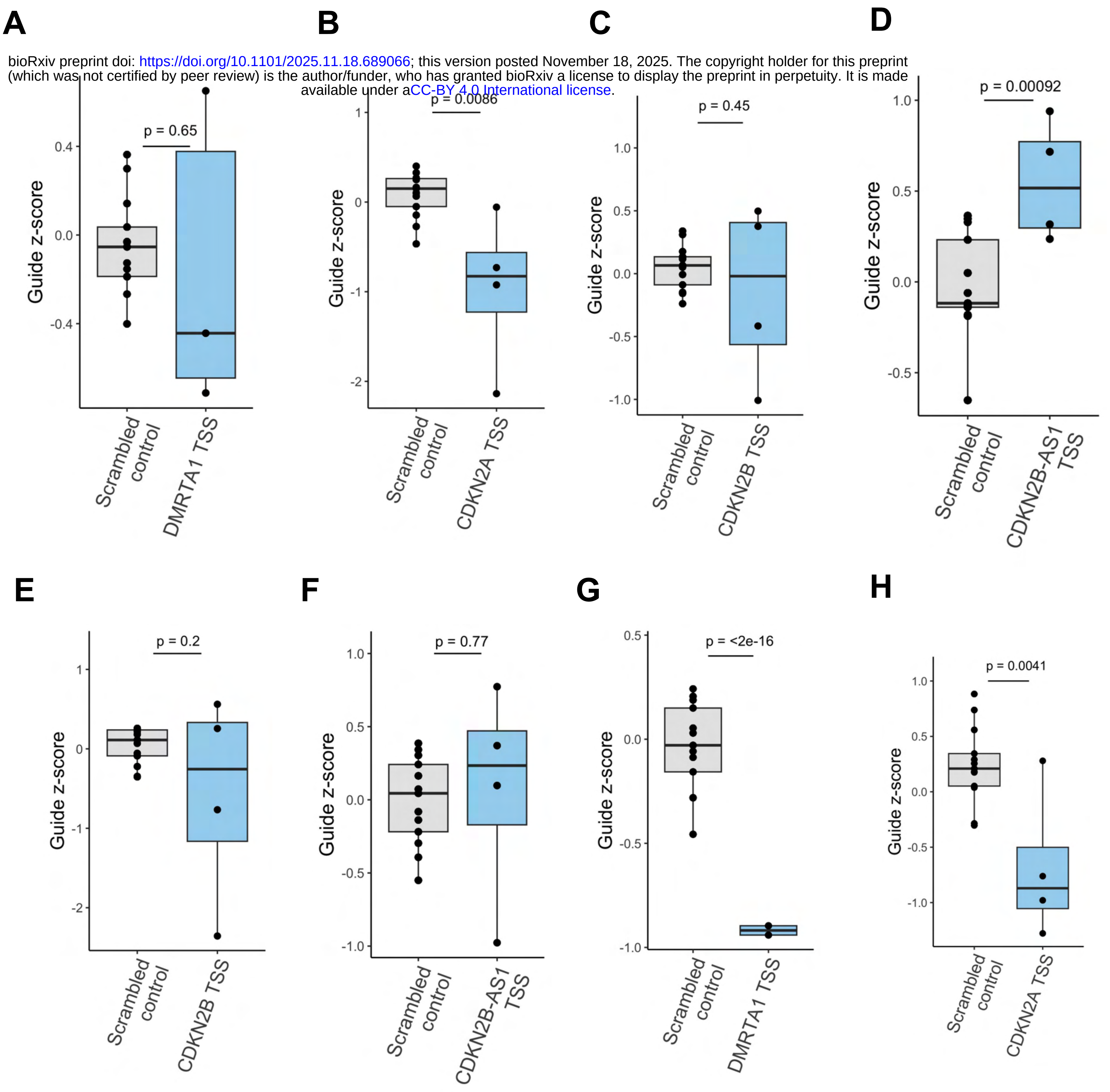

bioRxiv preprint doi: https://doi.org/10.1101/2025.11.18.689066; this version posted November 18, 2025. The copyright holder for this preprint (which was not certified by peer review) is the author/funder, who has granted bioRxiv a license to display the preprint in perpetuity. It is made available under aCC-BY 4.0 International license.

Figure S4. **Validation of TSS knockdown of cis-expressing *9p21.3* genes and evaluation of guide efficiency from z-score normalized CRISPRi-MAC-Seq gene expression data.**

(A-D) Box plot showing knockdown efficiency of *9p21.3* cis-expressing gene vascular fibroblast TSS knockdown efficiency. Analyzed by t-test with a significance threshold of P-value<0.05.

(E-H) Box plot showing knockdown efficiency of *9p21.3* cis-expressing gene vascular smooth muscle cell TSS knockdown efficiency. Analyzed by t-test with a significance threshold of P-value<0.05.

Figure S5

bioRxiv preprint doi: https://doi.org/10.1101/2025.11.18.689066; this version posted November 18, 2025. The copyright holder for this preprint (which was not certified by peer review) is the author/funder, who has granted bioRxiv a license to display the preprint in perpetuity. It is made available under aCC-BY 4.0 International license.

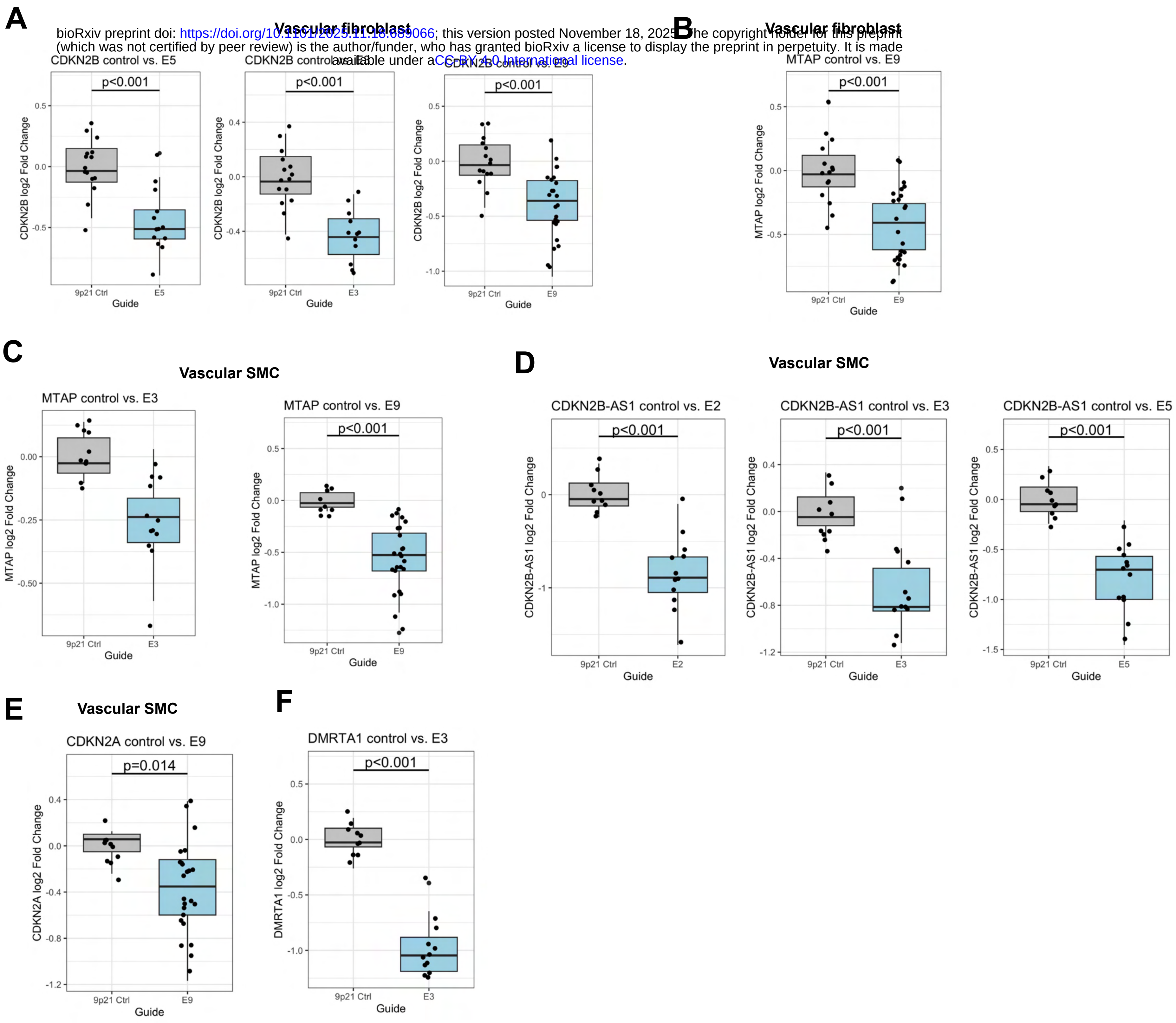

bioRxiv preprint doi: https://doi.org/10.1101/2025.11.18.689066; this version posted November 18, 2025. The copyright holder for this preprint (which was not certified by peer review) is the author/funder, who has granted bioRxiv a license to display the preprint in perpetuity. It is made available under aCC-BY 4.0 International license.

Figure S5. **Validation of *9p21.3* enhancer-2-gene (E2G) connection using a direct-lysis qPCR method (Invitrogen two-step Cells-to-Ct)**.

(A-B) Box plot showing effect of 9p21.3 enhancers on cis-gene expression in vascular fibroblasts. Analyzed by t-test with a significance threshold of $p<0.05$. Guides from the larger screen targeting enhancers E2, E3, E5, and E9 were independently cloned for this validation experiment.

(C-F) Box plot showing effect of 9p21.3 enhancers on cis-gene expression in vascular smooth muscle cells. Analyzed by t-test with a significance threshold of $p<0.05$. Guides from the larger screen targeting enhancers E2, E3, E5, and E9 were independently cloned for this validation experiment.

Figure S6

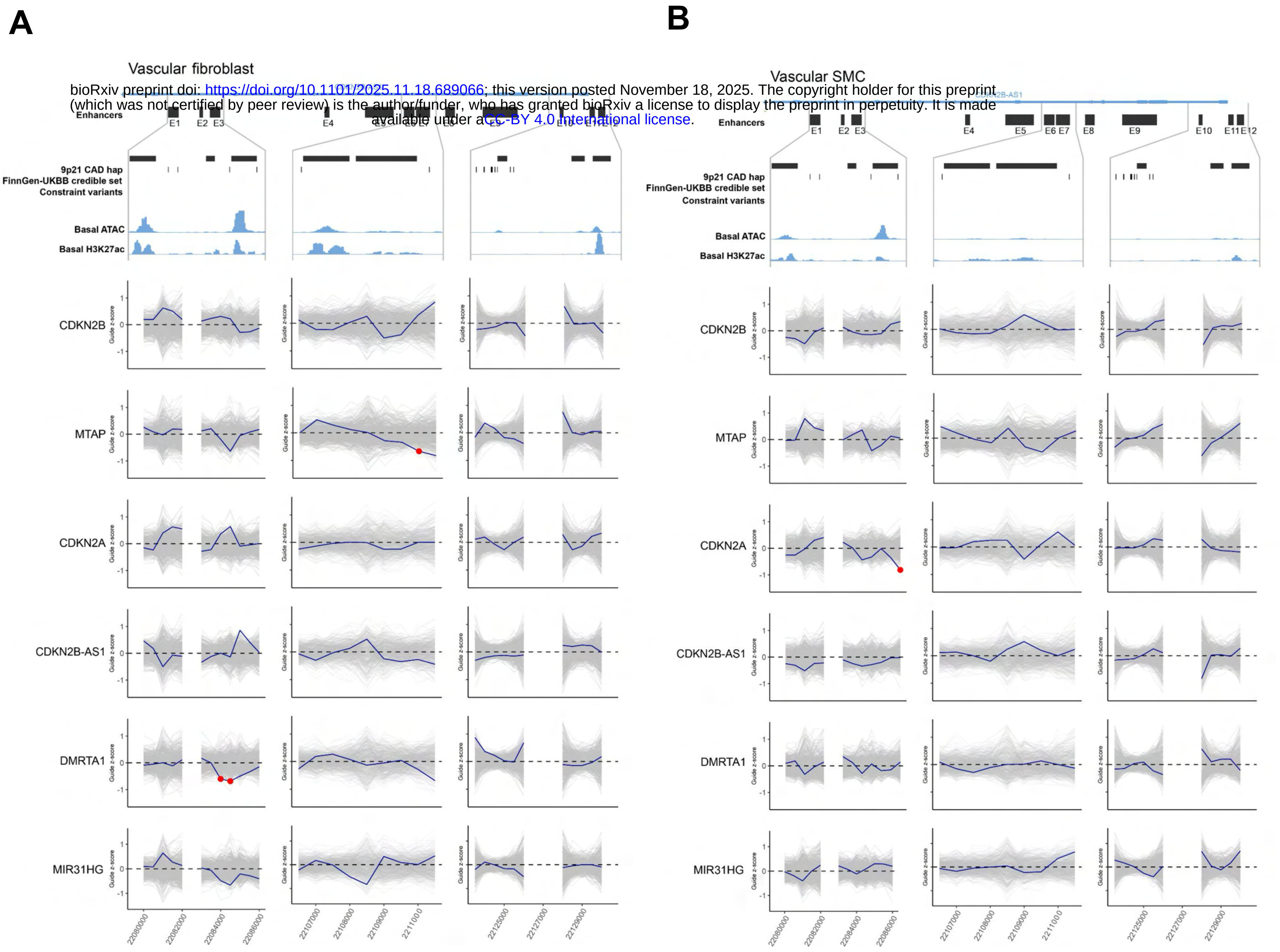

bioRxiv preprint doi: https://doi.org/10.1101/2025.11.18.689066; this version posted November 18, 2025. The copyright holder for this preprint (which was not certified by peer review) is the author/funder, who has granted bioRxiv a license to display the preprint in perpetuity. It is made available under aCC-BY 4.0 International license.

bioRxiv preprint doi: https://doi.org/10.1101/2025.11.18.689066; this version posted November 18, 2025. The copyright holder for this preprint (which was not certified by peer review) is the author/funder, who has granted bioRxiv a license to display the preprint in perpetuity. It is made available under aCC-BY 4.0 International license.

Figure S6. **Plot shows a sliding window approach that aggregates sgRNA transcriptional effects across the *9p21.3* annotated enhancers, including E1, E2, E3, E6, E7, E10, E11, and E12 in**

(A) vascular fibroblast,

(B) Vascular smooth muscle cells. To assign significance to observed data relative to the permutation background, a permutation z-score was calculated. A significance level of z-score=-1.96 was used to identify significantly repressed regions – illustrated as a red dot on the plots.

Figure S7

bioRxiv preprint doi: https://doi.org/10.1101/2025.11.18.689066; this version posted November 18, 2025. The copyright holder for this preprint (which was not certified by peer review) is the author/funder, who has granted bioRxiv a license to display the preprint in perpetuity. It is made available under aCC-BY 4.0 International license.

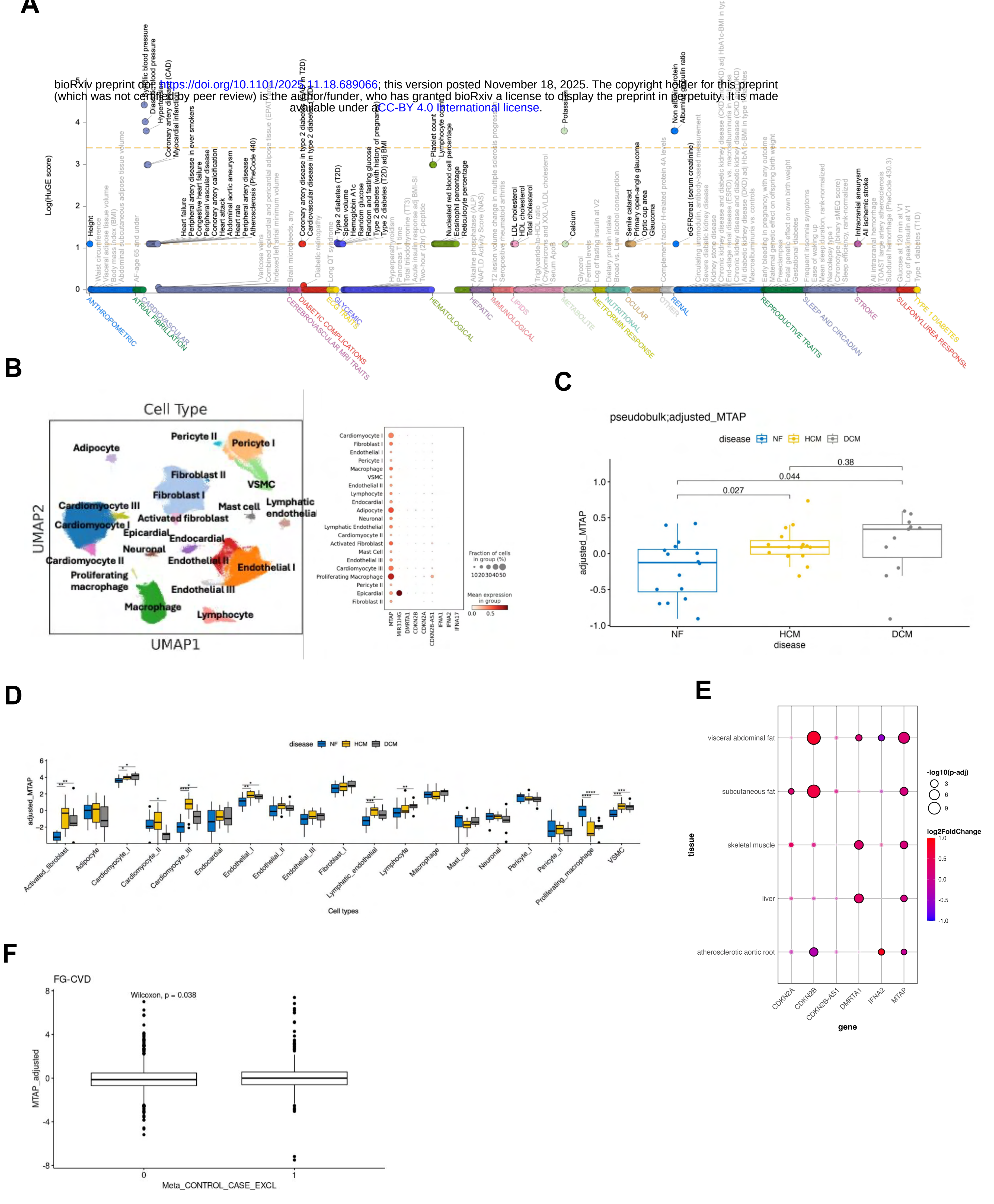

bioRxiv preprint doi: https://doi.org/10.1101/2025.11.18.689066; this version posted November 18, 2025. The copyright holder for this preprint (which was not certified by peer review) is the author/funder, who has granted bioRxiv a license to display the preprint in perpetuity. It is made available under aCC-BY 4.0 International license.

Figure S7. Translational relevance of MTAP in large-scale population

(A) left: Uniform manifold approximation and projection (UMAP) of 592,689 single-nucleus RNA profiles from left ventricle samples from 42 individuals [2]. Right: Expressions of the genes within the 9p21 locus in the 21 identified cell types. The dot plot highlights the expressions of MTAP across these cell types.

(B) *MTAP* expression was analyzed in non-failing (NF) participants and compared to patients with hypertrophic cardiomyopathy (HCM) and dilated cardiomyopathy (DCM) using box plots. Significant differences were observed when comparing pseudobulk expressions. Next panels show similar results using the expressions in the fibroblast and VSMC cell types. The trend of increased expression is not significant in fibroblast cells, however, a significant trend was observed in VSMC.

(C) Data from STARNET showing tissue-specific changes in 9p21 gene expression in CAD cases vs. control. *MTAP* expression was significantly changed across multiple tissues.

(D) Analysis of MTAP expression from the FinnGen Olink proteomics database showing a significant (p=0.038) increase in MTAP expression level in the circulating blood of CAD cases (mean=0.047, 1st quartile= -0.665, 3d quartile=0.520) relative to control (mean= -0.023, 1st quartile= -0.672, 3d quartile= 0.493).

(E) To demonstrate the gene prioritization is disease specific, we repeat the analysis using a schizophrenia GWAS. ZSTAT of none of *9p21* genes pass the 0.95th percentile cutoff.

Figure S8

bioRxiv preprint doi: https://doi.org/10.1101/2025.11.18.689066; this version posted November 18, 2025. The copyright holder for this preprint (which was not certified by peer review) is the author/funder, who has granted bioRxiv a license to display the preprint in perpetuity. It is made available under aCC-BY 4.0 International license.

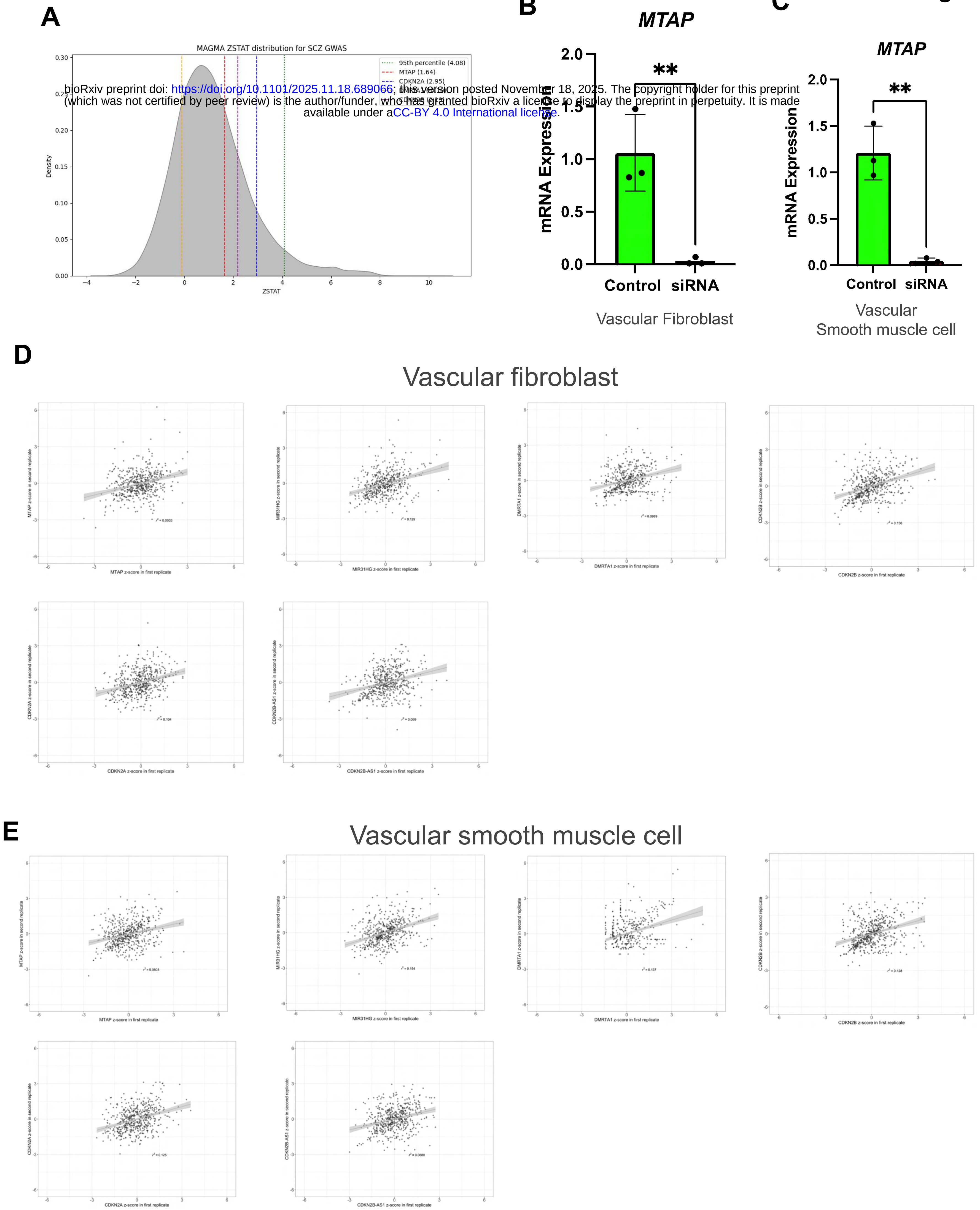

bioRxiv preprint doi: https://doi.org/10.1101/2025.11.18.689066; this version posted November 18, 2025. The copyright holder for this preprint (which was not certified by peer review) is the author/funder, who has granted bioRxiv a license to display the preprint in perpetuity. It is made available under aCC-BY 4.0 International license.

Figure S8. Convergent evidence for MTAP as a driver CAD gene.

(A) Density plot from MAGMA showing analysis on Schizophrenia disease trait .

(B-C) Validation of siRNA knockdown of *MTAP* in samples sent for bulk RNA-Seq

Figure S9

A

bioRxiv preprint doi: https://doi.org/10.1101/2025.11.18.689066; this version posted November 18, 2025. The copyright holder for this preprint (which was not certified by peer review) is the author/funder, who has granted bioRxiv a license to display the preprint in perpetuity. It is made available under aCC-BY 4.0 International license.

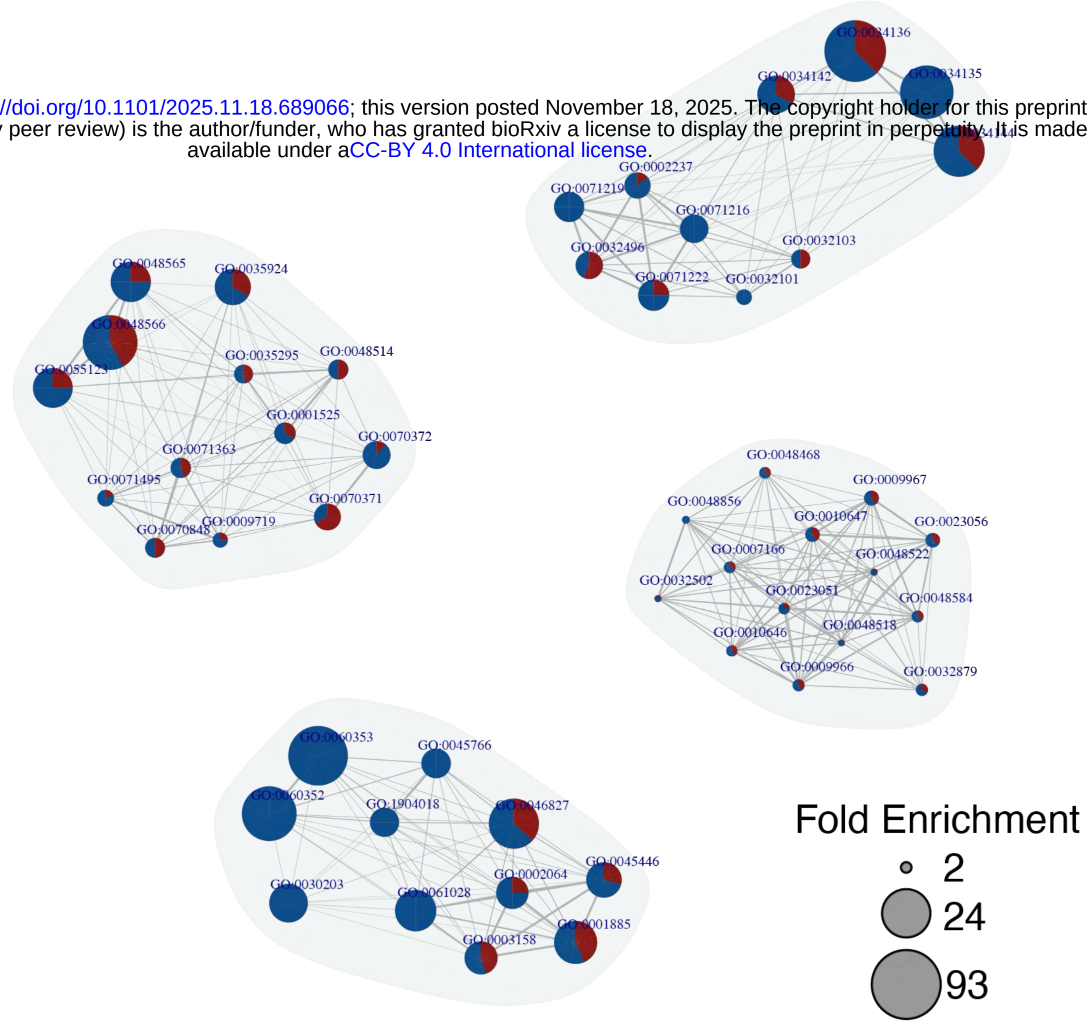

B

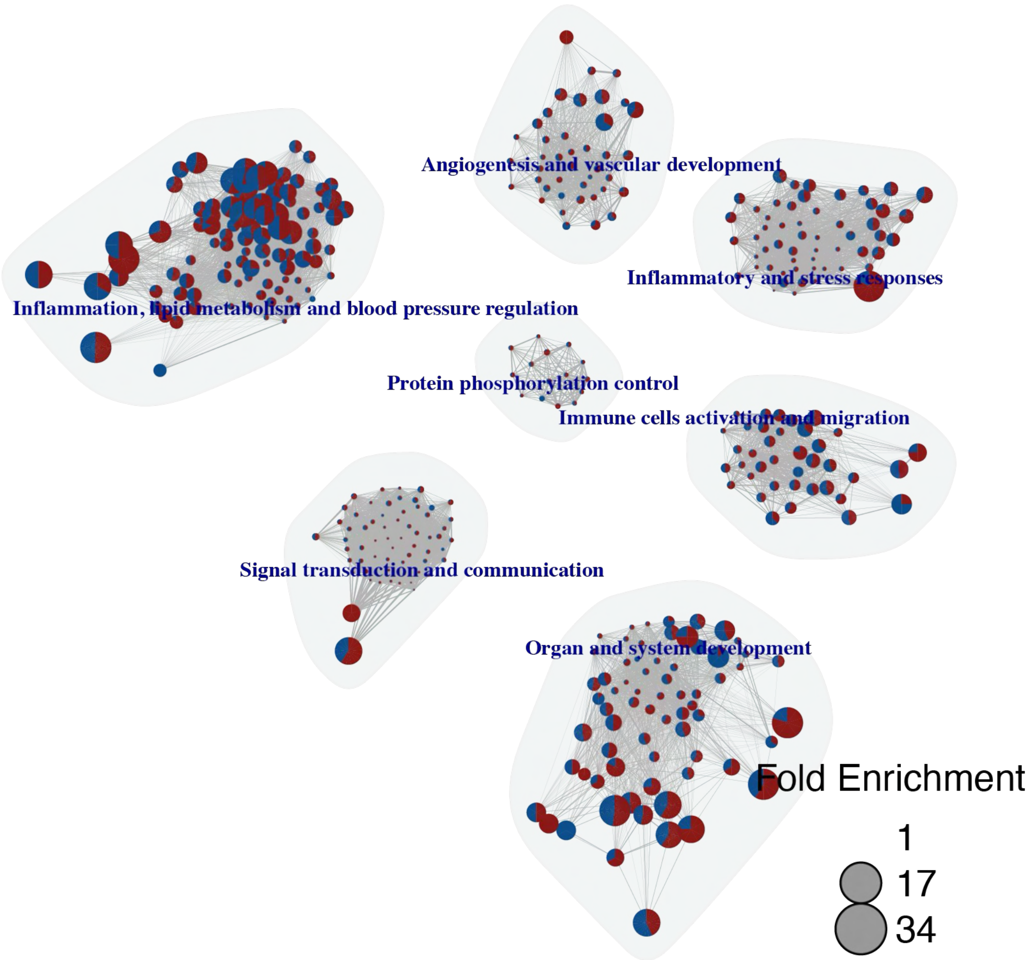

bioRxiv preprint doi: https://doi.org/10.1101/2025.11.18.689066; this version posted November 18, 2025. The copyright holder for this preprint (which was not certified by peer review) is the author/funder, who has granted bioRxiv a license to display the preprint in perpetuity. It is made available under aCC-BY 4.0 International license.

Figure S9. Network analysis of gene ontology pathways impacted by siMTAP knockdown in vascular fibroblast and smooth muscle cells, showing interconnected networks of disease-relevant modules in

(A) Vascular smooth muscle cells.

(B) Vascular Fibroblasts.

Figure S10

bioRxiv preprint doi: https://doi.org/10.1101/2025.11.18.689066; this version posted November 18, 2025. The copyright holder for this preprint (which was not certified by peer review) is the author/funder, who has granted bioRxiv a license to display the preprint in perpetuity. It is made available under aCC-BY 4.0 International license.

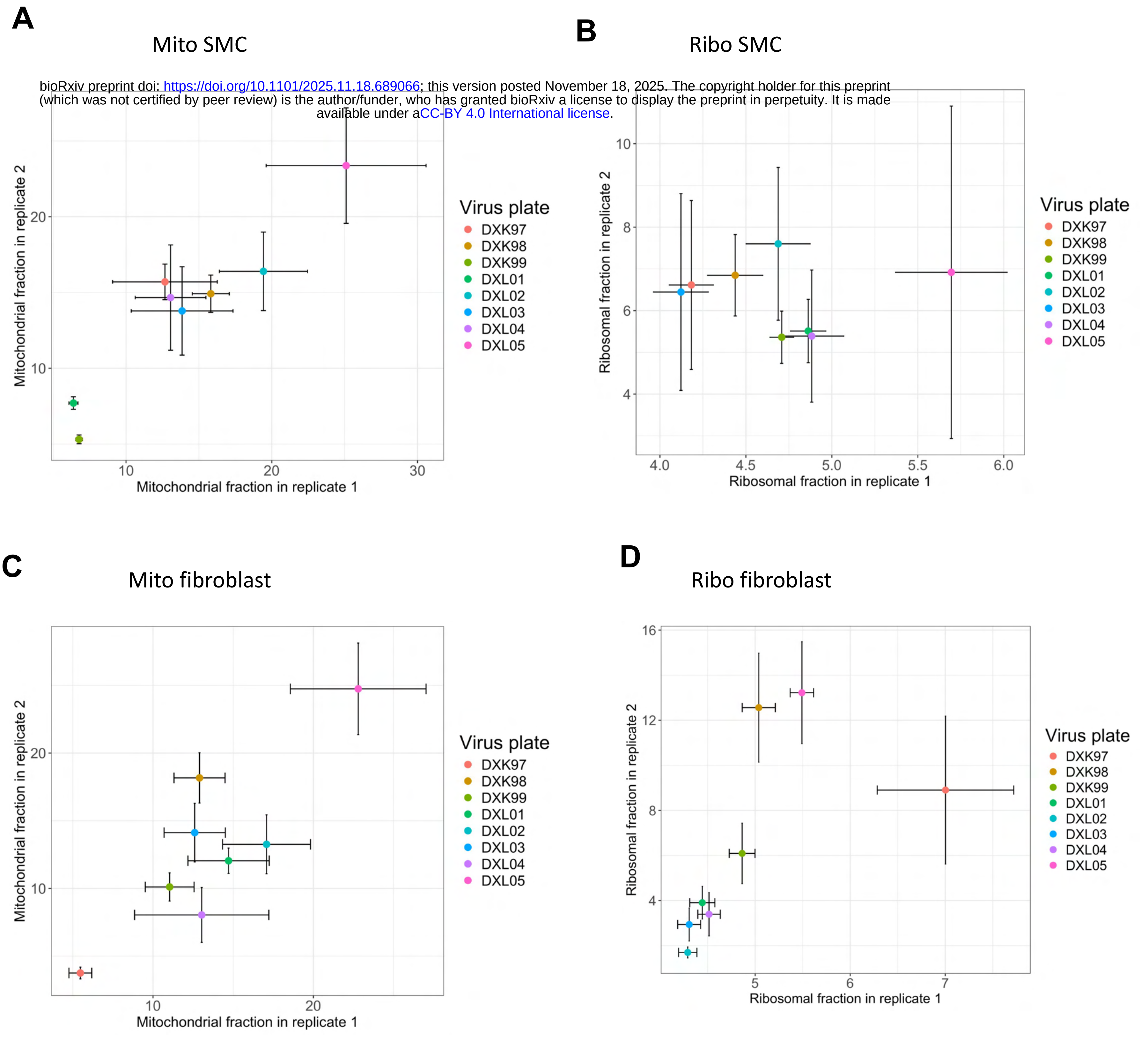

bioRxiv preprint doi: https://doi.org/10.1101/2025.11.18.689066; this version posted November 18, 2025. The copyright holder for this preprint (which was not certified by peer review) is the author/funder, who has granted bioRxiv a license to display the preprint in perpetuity. It is made available under aCC-BY 4.0 International license.

Figure S10. QC showing overall mitochondrial and ribosomal gene fraction for our CRISPRI-MAC-seq screen. For all plots, the x-axis shows the first biological replicate for each of 8 MAC-Seq arrayed plates, and the y-axis shows the second replicate.

(A) Mitochondrial fraction (%) by plate with standard deviation in vascular smooth muscle cells.

(B) Ribosomal fraction (%) by plate with standard deviation in vascular smooth muscle cells.

(C) Mitochondrial fraction (%) by plate with standard deviation in vascular fibroblasts.

(D) Ribosomal fraction (%) by plate with standard deviation in vascular fibroblasts.

bioRxiv preprint doi: https://doi.org/10.1101/2025.11.18.689066; this version posted November 18, 2025. The copyright holder for this preprint (which was not certified by peer review) is the author/funder, who has granted bioRxiv a license to display the preprint in perpetuity. It is made available under aCC-BY 4.0 International license.

Figure S11

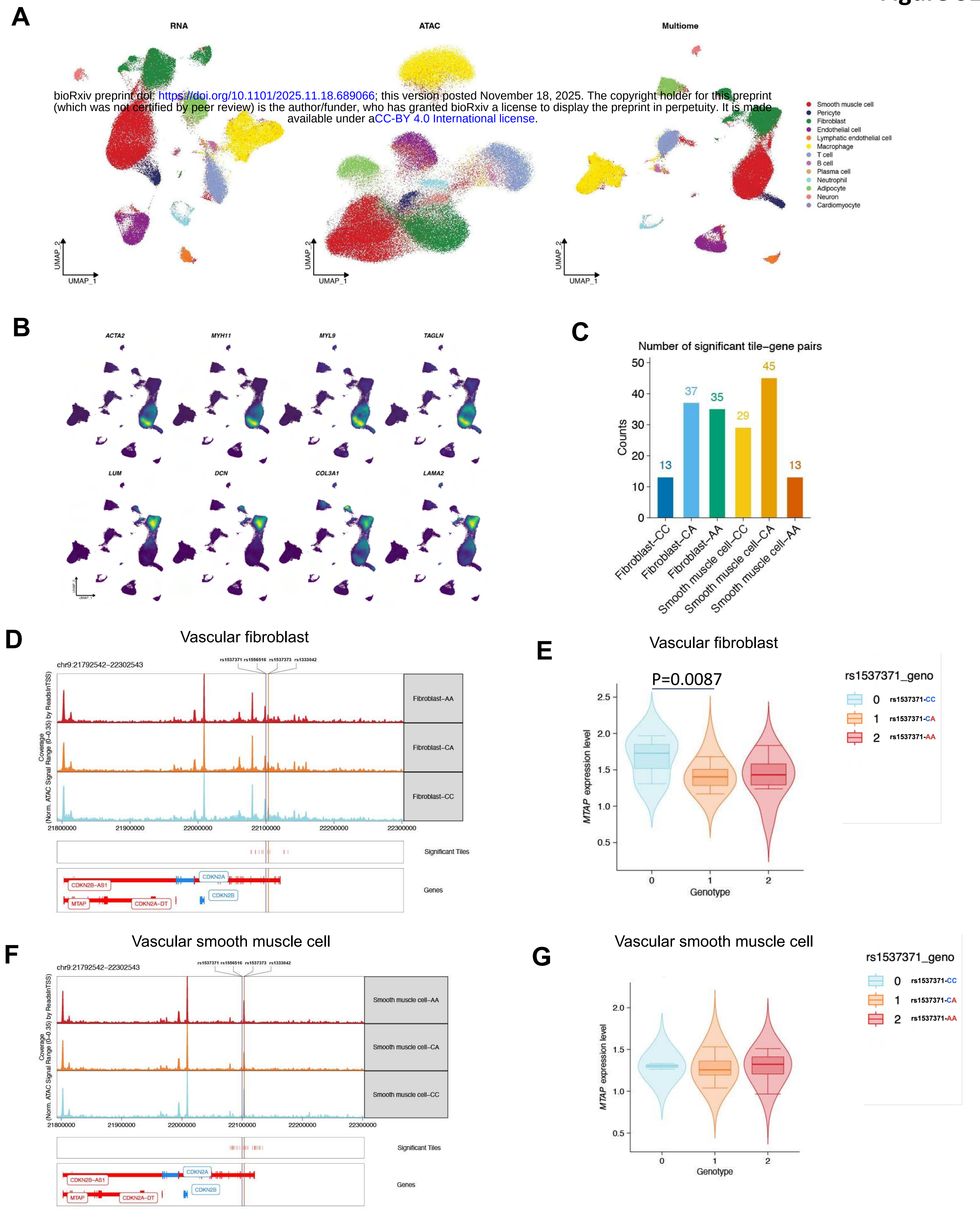

bioRxiv preprint doi: https://doi.org/10.1101/2025.11.18.689066; this version posted November 18, 2025. The copyright holder for this preprint (which was not certified by peer review) is the author/funder, who has granted bioRxiv a license to display the preprint in perpetuity. It is made available under aCC-BY 4.0 International license.

Figure S11. scMultiome analysis of Left anterior descending (LAD) coronary artery

(A) Uniform manifold approximation and projection (UMAP) of scMultiome (scATAC-seq + scRNA-seq) data from Human left anterior descending (LAD) coronary artery, with cells colored by cell types

(B) Density plot showing cell type specific markers for vascular smooth muscle cell (*ACTA2*, *MYH11*, *MYL9*, and *TAGLN*) and vascular fibroblasts (*LUM*, *DCN*, *COL3A1*, and *LAMA2*).

(C) Bar plot showing significant *9p21.3* tile-to-gene pair counts of *9p21.3* cis-expressing genes (*MTAP*, *DMRTA1*, and *CDKN2B-AS1*) stratified by genotype at rs1537371 in vascular fibroblasts and smooth muscle cells

(D) Genomic track shows accessibility of *9p21.3* locus at rs1537371 SNP in vascular fibroblast.

(E) Allelic dependent pseudobulk mRNA expression of *MTAP* in vascular fibroblast

(F) Genomic track shows accessibility of *9p21.3* locus at rs1537371 SNP in vascular smooth muscle.

(G) Allelic dependent pseudobulk expression of MTAP in vascular smooth muscle

bioRxiv preprint doi: https://doi.org/10.1101/2025.11.18.689066; this version posted November 18, 2025. The copyright holder for this preprint (which was not certified by peer review) is the author/funder, who has granted bioRxiv a license to display the preprint in perpetuity. It is made available under aCC-BY 4.0 International license.

Supplementary Tables (available as spreadsheet workbook)

Table S1. PheWAS data from FinnGen freeze 13, shown in panel 1A, for the *9p21* tag SNP rs4977574. *9p21.3* is robustly associated with coronary artery disease and other vascular disease traits.

Table S2. The haplotype table, sourced from HaploReg (v4.2)[3], presents variant information in linkage disequilibrium with the primary *9p21* variant.

Table S3. LD-score regression heritability analysis of vascular disease and control (left-handedness and schizophrenia) traits in vascular cells under nutrient and inflammatory stimulatory conditions.

Table S4. Differential H3k27ac peaks within the *9p21* locus for 4 vascular cell lines, vascular fibroblasts, smooth muscle cells, endothelial cells, and adipose pericytes. Differential peaks were calculated using DiffBind, with a genome-wide significance level of FDR=0.05.

Table S5. Potentially cis-regulated target genes within the *9p21* TAD, gene function, and distance from each gene TSS to the locus.

Table S6. CRISPRi-MAC-Seq *9p21* library screen structure. The library contains 417 guides, including locus-targeting controls, scrambled controls, TSS, enhancer, and SNP-targeting guides. This table included guide position, sequence, the target the guide was designed for, and an additional column to note enhancer overlap of SNP-associated guides.

Table S7: Proportion of the 417 guides in the screen targeting each control type or genomic feature. Also shown in Figure 2c.

Table S8. Constraint analysis of the *9p21.3* locus. Constraint values are calculated for 1kb blocks using Gnocchi, and represent a constraint z-score relative to the whole genome. Regions that overlap SNPs from the CAD GWAS haplotype are indicated; a Gnocchi z-score of ≥ 2 was used as a threshold for significant constraint.

Table S9. WASP [4] was used to examine the link between heterozygous *9p21* variants and regulatory targets. A cutoff of $P < 0.05$ was considered to identify significant variant effects of individual *9p21.3* haplotype variants. Subsequently, the sum of the read counts corresponding to significant effects was used to represent the effect of the *9p21.3* haplotype on individual genes.

Table S10. HuGE-AMP data from the Common Metabolic Diseases Knowledge Portal, using genetic evidence from rare and common genetic variation to implicate MTAP in vascular disease processes (accompanies figure S8).

bioRxiv preprint doi: https://doi.org/10.1101/2025.11.18.689066; this version posted November 18, 2025. The copyright holder for this preprint (which was not certified by peer review) is the author/funder, who has granted bioRxiv a license to display the preprint in perpetuity. It is made available under aCC-BY 4.0 International license.

Table S11. Rare-variant analysis of potential *9p21.3* targets indicated nominal significance of *MTAP* (p=0.0919) in disease phenotypes.

Table S12. International Mouse Phenotyping Consortium (IMPC) database showing phenotypes associated with *Mtap* haploinsufficiency

Table S13. Gene prioritization using MAGMA. This table shows MAGMA gene-based Z-scores and P-value for CAD GWAS.

Table S14. Schizophrenia-risk GWAS gene-trait association for *9p21.3* genes. Intended as a control trait for CAD-risk, only one *9p21.3* gene, *MIR31HG*, ranked above the 95th percentile.

Table S15. DEGs (generated using DESeq2) for siMTAP knockdown relative to the non-targeting control in vascular smooth muscle cells under basal conditions.

Table S16. Gene Ontology (GO) enrichment of the differentially expressed genes (DEG) in VSMC was performed to identify functional clusters of enriched pathways. This table presents the mapping of pathways to clusters and provides functional annotations for each cluster.

Table S17. Network properties for functional clusters enriched in differentially expressed genes in vascular smooth muscle cells.

Table S18. DEGs from comparing basal vs MTAP perturbation in vascular fibroblasts

Table S19. Gene Ontology (GO) enrichment of the differentially expressed genes (DEG) in fibroblast was performed to identify functional clusters of enriched pathways. This table presents the mapping of pathways to clusters and provides functional annotations for each cluster.

Table S20. Network properties for functional clusters enriched in differentially expressed genes in vascular fibroblasts .

Table S21. Total number of morphological extracted features captured by LypocyteProfiler on vascular fibroblast and smooth muscle cells

Table S22. Pearson correlation of LipocyteProfilerfeatures in vascular fibroblasts across individual replicates of treatment conditions.

Table S23. Pearson correlation of LipocyteProfilerfeatures in vascular SMCs across individual replicates of treatment conditions.

bioRxiv preprint doi: https://doi.org/10.1101/2025.11.18.689066; this version posted November 18, 2025. The copyright holder for this preprint (which was not certified by peer review) is the author/funder, who has granted bioRxiv a license to display the preprint in perpetuity. It is made available under aCC-BY 4.0 International license.

Table S24. LipocyteProfiler feature comparison between gene control and siMTAP treatment in vascular fibroblasts for basal and TGF-β stimulated conditions

Table S25. LipocyteProfiler feature comparison between gene control and siMTAP treatment in vascular fibroblasts for basal and TGF-β stimulated conditions.

Table S26. MAC-seq codes used for multiplex library preparation and sequencing.

Table S27. Statistical analysis (Wilcoxon) of pseudobulked, annotated cell types from a human dilated and hypertrophic cardiomyopathy dataset.

Table S28. Quantification and statistical details for image analysis QC

Table S29. Primers used for qPCR analysis of *9p21.3* genes.

Table S30. Patient demographies for sc-Multiome

Table S31. Output from SCENT analysis for vascular fibroblasts and smooth muscle cells

Table S32. Genome-wide distribution of MAGMA gene-based Z-scores and P-value for T2D GWAS
